# DEHP mediates drug resistance by metabolic reprogramming in colorectal cancer cells

**DOI:** 10.1007/s11356-022-25110-1

**Published:** 2023-02-07

**Authors:** Yue Wu, Ruijie Lu, Yujie Lin, Jinjin Wang, Zijian Lou, Xiaochun Zheng, Ling Zhang, Ruolang Pan, Gang Lu, Qingxia Fang

**Affiliations:** 1 Center for Clinical Pharmacy, Cancer Center, Department of Pharmacy, Zhejiang Provincial People’s Hospital (Affiliated People’s Hospital, Hangzhou Medical College), Zhejiang Province, Hangzhou, 310014 China; 2 The Second Clinical Medical College, Wenzhou Medical University, Wenzhou, China; 3 Institute for Cell-Based Drug Development of Zhejiang Province, Hangzhou, China; 4 Key Laboratory of Cell-Based Drug and Applied Technology Development in Zhejiang Province, Hangzhou, China

**Keywords:** Diethylhexyl phthalate, Colorectal cancer, Metabolic reprogramming, Fluorouracil, Oxidative phosphorylation, Glycolysis

## Abstract

Long-term exposure to diethylhexyl phthalate (DEHP), an endocrine-disrupting chemical (EDCs) and plasticizer widely used in consumer products, has been reported to be significantly positively correlated with increased risks of different human diseases, including various cancers, while the potential effect of DEHP on colorectal cancer progression was little studied. In the present study, we showed that DEHP could trigger the metabolic reprogramming of colorectal cancer cells, promote cell growth and decrease fluorouracil (5-FU) sensitivity. Mechanistic studies indicated that DEHP could reduce glycolysis activity and increase oxidative phosphorylation (OXPHOS) in SW620 cells. In addition, in vivo experiments showed that DEHP promoted tumorigenic progression and decreased survival time in mice. Collectively, our findings suggest that DEHP may be a potent risk factor for colorectal cancer development.

## Introduction

Phthalates (PAEs) are the most commonly used plasticizers in plush toys, flooring, medical devices, cosmetics, air fresheners, etc. (Schettler 2006). PAEs bind non-covalently to plastics, are readily released from plastic materials, and are harmful by polluting air, soil, water, and even food (Jebara et al. 2021). Thus, PAEs have been listed as top priority environmental pollutants by the US Environmental Protection Agency and the European Union (Promtes et al. 2019, Gani and Kazmi 2020). Di(2-ethyl)hexyl phthalate (DEHP) is a typical PAEs, which accounts for more than half of the global usage of PAEs (Wang et al. 2019, Li et al. 2021). DEHP concentrations in the environment have gradually increased along with the development of global manufacturing. Furthermore, DEHP can accumulate in the human body via many ways, such as through water, food, airborne particles, and skin protection products (Wang et al. 2019) .

In recent decades, with the widespread use of DEHP-containing products, the toxicity of DEHP has caused continued concern in the scientific community. Studies have reported that long-term exposure to DEHP will have various effects on the human body (Stenz et al. 2019, Trnka et al. 2021, Zhao et al. 2021). DEHP may affect the liver, kidney, cardiovascular system, and reproductive organs, increase fetal mortality, cause malformations and low birth weight, etc. (Camacho et al. 2020, Chang et al. 2021, Trnka et al. 2021). In recent years, there have been frequent reports on the effects of long-term exposure to DEHP on the progression of various types of tumors (Chou et al. 2018, Zhang et al. 2021). Most of these studies focus on liver, breast, and prostate cancers (Chou et al. 2018, Camacho et al. 2020). However, little is known about whether DEHP might affect colorectal cancer (CRC) growth and chemoresistance. Dikmen et al. demonstrated induction of cancer cells proliferation by low-dose phthalate mixtures in vitro (Yurdakok Dikmen et al. 2015). A recent study also proved that urinary MEHP concentrations, a potent metabolite of DEHP, were significantly higher in CRC patients than in the healthy participants (Su et al. 2021). In addition, it is reported that 5-FU is one of the most commonly used chemotherapeutic drugs for the treatment of CRC (Wolpin and Mayer 2008). However, there are few research on the effects of DEHP on colon cancer cells proliferation, metabolic function, and drug resistance. The present study aimed to investigate the potential effects of DEHP on the 5-FU sensitivity of colorectal cancer cells. Moreover, the potential mechanisms underlying the DEHP-regulated progression of colorectal cancer were also investigated.

## Materials and methods

### Cell lines and culture conditions

SW620 cells were purchased from the American Type Culture Collection (ATCC, USA) and were cultured in DMEM (Sigma-Aldrich) supplemented with 10% fetal bovine serum (FBS, Gibco) in a humidified atmosphere of 5% CO_2_ and 95% air. In vitro long-term exposure of SW620 cells was performed by administering 1 μM DEHP for over 2 months. The medium was replaced daily to ensure the DEHP concentration. Cells were harvested during the log phase of growth and were seeded at a density of 0.2 million cells per mL.

### Animal experiments

Fourteen male nu/nu nude mice at 12 weeks of age were purchased from Beijing Vital River Laboratory Animal Technology Co. All mice were housed in an air-conditioned semipathogen-free room with a 12-h light/dark cycle with free access to water and chow. All experimental procedures were performed according to the “Guidelines for the Care and Use of Laboratory Animals” approved by the local Institutional Animal Care and Use Committee. Mice were randomly divided into two groups (*n* = 7), each receiving100 µL subcutaneous injection containing 1 × 10^6^ DEHP-pretreated SW620 or parental SW620 cells. Tumor length and width were detected by calipers, and tumor volume was calculated using the formula: length × width^2^) × 0.5.

### Apoptosis analysis

SW620 cells were treated with 1.5 μg/ml of 5-FU (ST1060, Beyotime) for 48 or 72 h, and the apoptotic cell numbers were analyzed using Annexin V-propidium iodide binding assays (C1062M, Beyotime). Flow analyses were performed using a BD FACSCalibur™ Flow Cytometer. The percentages of Annexin V-positive cells and the absolute cell counts were determined using counting beads assays (ThermoFisher C36950).

### Glucose uptake

Glucose uptake was determined by measuring the uptake of 2-[14C]-deoxy-D-glucose (2-DG) (ab235976) according to the introduced protocol. In brief, 5 × 10^4^ cells were seeded in a 96-well plate with 100 µL culture medium. The next day, the culture medium was replaced to a glucose-free culture medium. Then, add 2-NBDG to a final concentration of 100–200 µg/mL in the glucose-free medium. Sixteen hours later, aspirate the supernatant and add 200 µL of cell-based assay buffer to each well. Centrifuge the plate for 5 min at 400 g at room temperature. Add 100 µL of cell-based assay buffer to each well. Finally, NBDG taken up by the cells can be detected with fluorescent filters usually designed to detect fluorescein (excitation/emission = 485/535 nm).

### Determination of glucose consumption in culture medium

Cell culture media were collected 24 h after incubation. Glucose consumption in the culture medium was determined using Glucose (GO) Assay Kit (GAGO20, Sigma-Aldrich) following the manufacturer’s instructions.

### Western blotting analysis

Western blotting analysis was performed using a standard protocol. Antibodies used in this study include anti-HK1 and anti-HK2 from Santa Cruz Biotechnology and anti-PKM2 from Proteintech. Immunoreactive proteins were visualized using a chemiluminescent immunodetection system (ChemiDoc XRS). ImageJ was employed to analyze the grayscale values using anti-actin (byotiome) as housekeeping control.

### Measurement of the extracellular acidification rate (ECAR), oxidative phosphorylation (OCR), and adenosine triphosphate (ATP)

Cellular ECAR and OCR alternations were determined with the Seahorse XF96 Flux Analyzer (Seahorse, Agilent) in real-time according to the manufacturer’s instructions. The cellular ATP levels were measured by standard assay kits purchased from Beyotime according to the manufacturer’s instructions.

### Statistical analysis

All analyses were performed using Prism GraphPad 6.00, and the data are presented as means ± standard deviations from at least three independent tests. Statistical differences between the two groups were evaluated by Student’s *t*-tests. Statistical significance was defined as *P* < 0.05.

## Results

### DEHP mediates drug resistance in SW620 cells

**Fig. 1:**
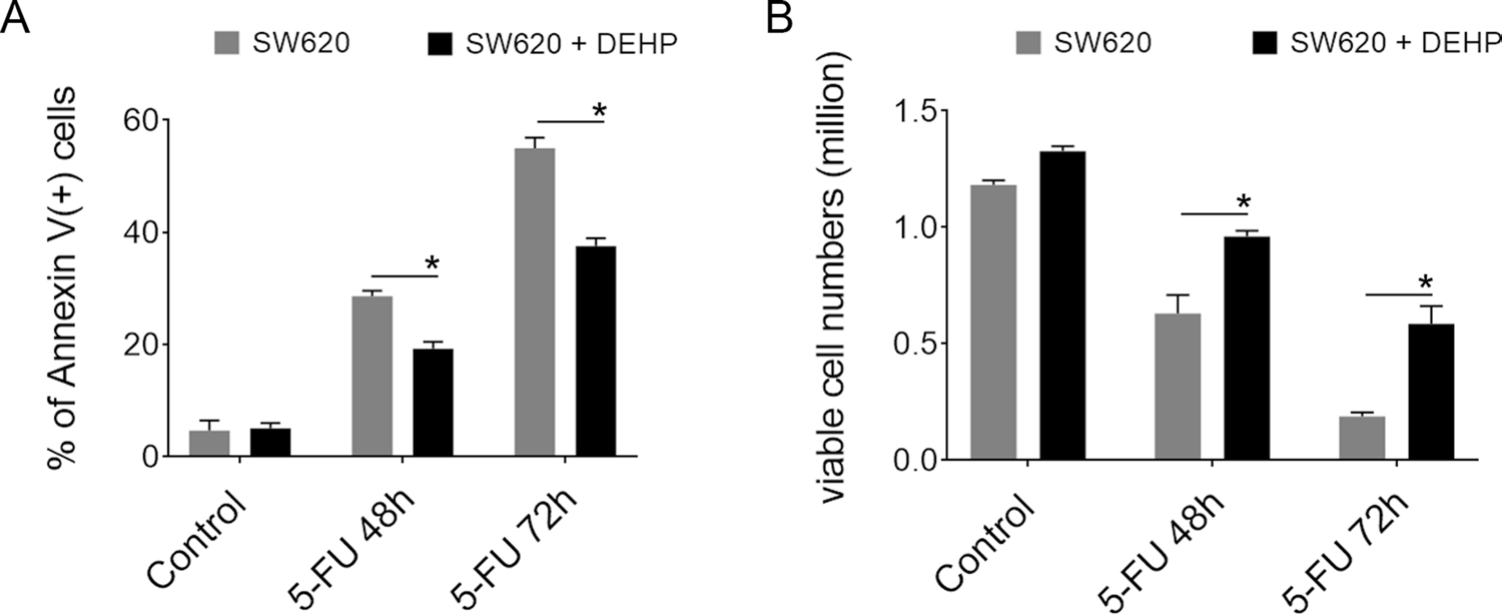
Treatment of SW620 cells with DEHP leads to increased resistance to 5-FU. DEHP-pretreated and parental SW620 cells were incubated with 1.5 μg/ml 5-FU. **A** The apoptosis ratios (percent of Annexin V.^+^ cells) at different time points were determined using flow cytometry. **B** The numbers of viable cells were calculated. (*n* = 3, *P < 0.05)

The DEHP-pretreated cells and the parental SW620 cells were cultured in a growth medium with or without 1.5 μg/ml 5-FU for 3 days. As shown in Fig. 1A, following 5-FU treatments, apoptosis was induced in parental SW620 cells at approximately 28.1 ± 1.9% at 48 h and 55.2 ± 3.7% at 72 h. Of note, the apoptosis ratios were significantly reduced by ~ 30% in DEHP-pretreated SW620 cells (19.4 ± 1.5% at 48 h and 38.2 ± 3.1% at 72 h). Furthermore, the number of viable cells was determined by flow cytometry (Fig. 1B). Consistent with the results of annexin-V/PI analysis, the number of viable cells was significantly higher in the DEHP-pretreated group compared with the control group (960,700 ± 24,580 vs. 630,300 ± 78,620 at 48 h; 585,700 ± 76,290 vs. 188,300 ± 17,210 at 72 h; all *P* < 0.05).

### Glycolysis activity is decreased in DEHP-pretreated SW620 cells

**Fig. 2:**
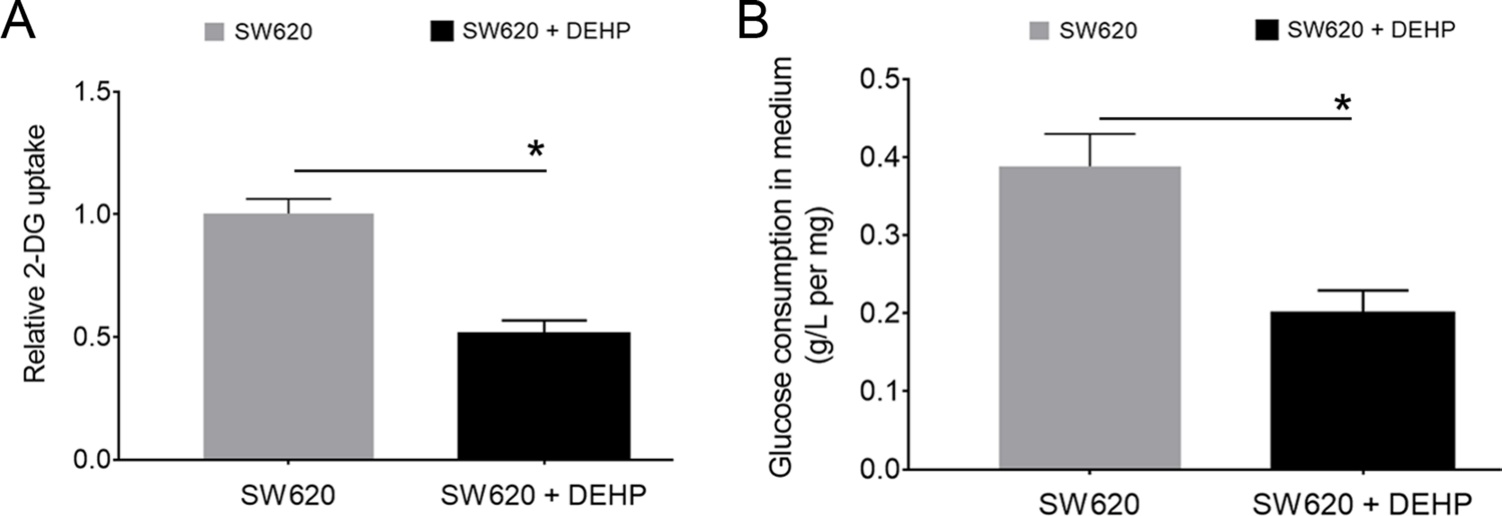
Decreased glycolysis activity in DEHP-pretreated SW620 cells. **A** Relative glucose consumption by 2-DG uptake assay. **B** Glucose contents were determined in culture media from two groups of cells incubated for 24 h. The results were normalized by total protein amounts. (*n* = 3, *P < 0.05 vs. 2-DG uptake in parental cell line)

To explore the mechanism of DEHP-mediated chemoresistance, we examined glucose metabolism in these SW620 cells. As shown in Fig. 2A, the results of the 2-DG uptake assay showed that glucose uptake in these DEHP-pretreated cells was significantly reduced (~ 49.8 ± 3.7% lower) compared with the SW620 parental cells. Moreover, the glucose consumption assay also confirmed that the amount of glucose that was left in the culture medium was statistically higher in the DEHP-pretreated group than in the parental control group (Fig. 2B).

**Fig. 3:**
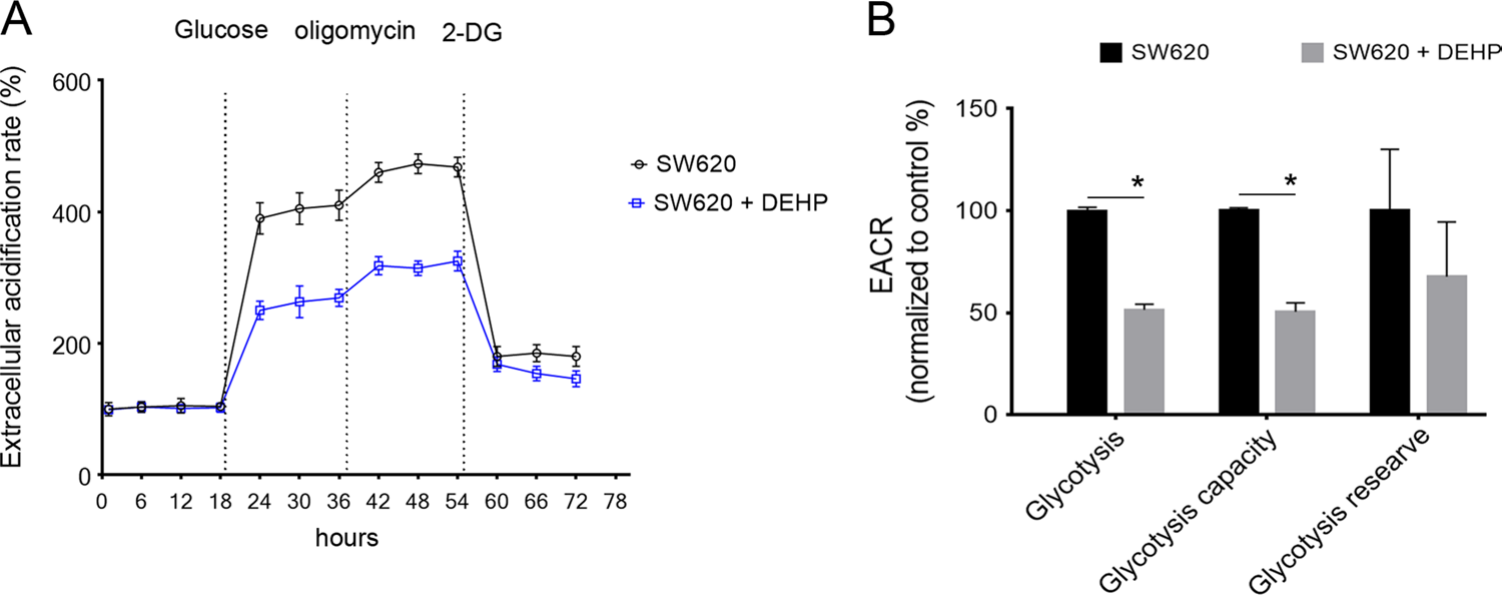
Change of ECAR in DEHP-pretreated SW620 cells. **A** ECAR values were obtained from the glycolysis stress test using the Agilent Seahorse XF technology. Glucose, oligomycin, and 2-DG were added at indicated time points. **B** The glycolysis, glycolysis capacity, and glycolysis reserve were calculated. (*n* = 3, *P < 0.05)

**Fig. 4:**
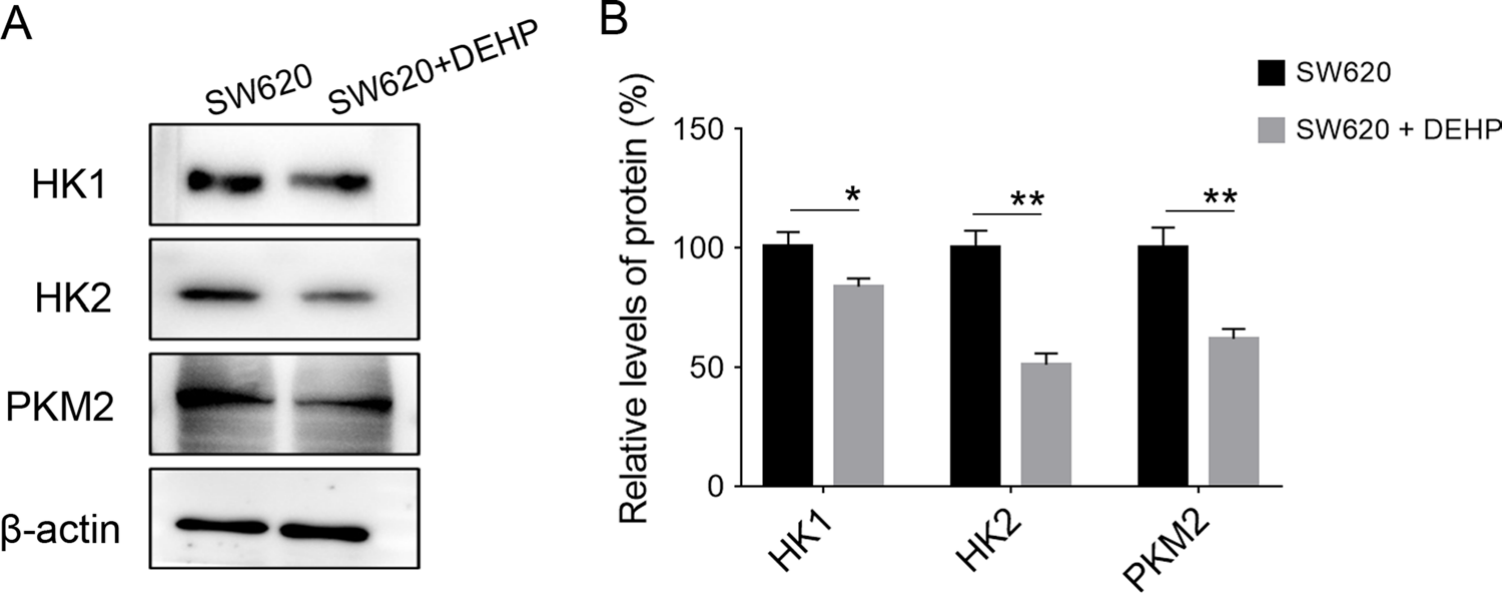
Decreased expression of glycolysis enzymes in DEHP-pretreated SW620 cells. **A** Expression levels of the representative glycolysis enzymes HK1, HK2, and PKM2 were determined using western blot and were normalized to those of ACTIN. **B** Relative expression levels from 3 replicate experiments are quantitated and subjected to statistical analysis. (*n* = 3, *P < 0.05; **P < 0.01)

Furthermore, cell metabolism measurements were performed using the XF96 Extracellular Flux analyzer with a glycolysis stress kit. The results suggested that metabolic flexibility was discrepant between the two groups of cells. DEHP-pretreated SW620 cells exhibited a reduced extracellular acidification rate (ECAR), representing glycolysis (Fig. 3). These data indicated that DEHP administration leads to a significantly decreased in both glycolysis and glycolysis capacity. We determined whether the expression levels of representative enzymes involved in glycolysis were regulated. As shown in Fig. 4, the protein level of HK1 was ~ 16.3 ± 3.5% lower in DEHP-pretreated cells compared with the control. More strikingly, the protein levels of HK2 and PKM2 decreased to ~ 50% of the parental cells. These metabolic assays indicated that glycolysis capacity was decreased in DEHP-pretreated cells with upregulated chemoresistance.

### Mitochondrial OXPHOS function is upregulated in DEHP-pretreated SW620 cells

**Fig. 5:**
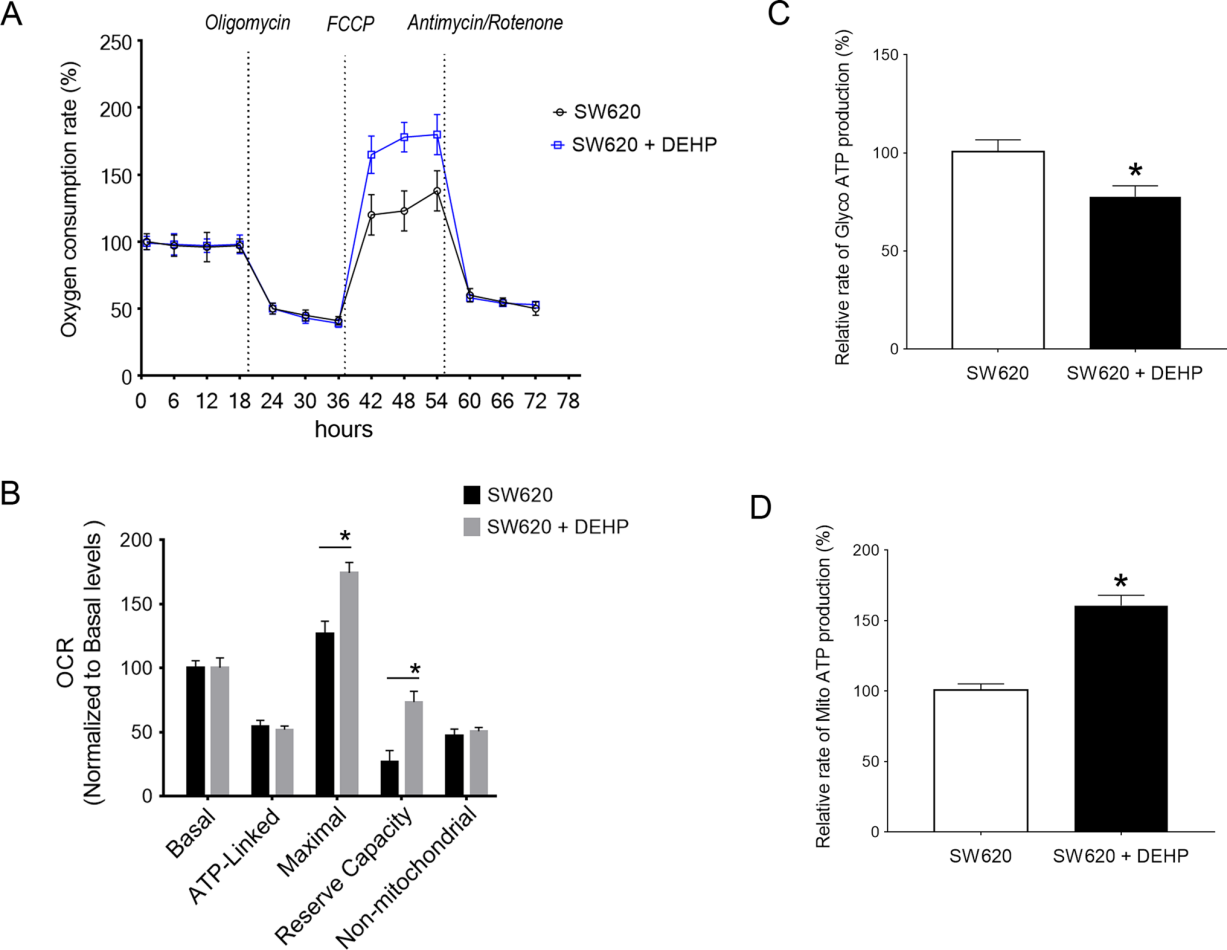
Enhanced OXPHOS activity in DEHP-pretreated SW620 cells. **A** OCR values were measured using the Agilent Seahorse XF technology. Oligomycin, FCCP, rotenone, and antimycin A were added at indicated times to determine different parameters of mitochondrial functions. **B** Graphs presented the ATP-linked OCR, proton leak OCR, maximal OCR, reserve capacity, and non-mitochondrial OCR. **C** and **D** ATP production rate from glycolysis and OXPHOS was simultaneously determined in two groups of cells. (*n* = 3, *P < 0.05)

To further evaluate the effect of DEHP administration in SW620 cells, the cellular oxygen consumption rates (OCRs) were also determined using a seahorse instrument after the sequential addition of oligomycin, FCCP, rotenone, and antimycin. As shown in Fig. 5A, the two OCR curves closely overlapped before the addition of FCCP, indicating no significant difference in basal OCR and ATP-linked OCR between the two groups of cells. Notably, the maximal OCR and reserve capacity in DEHP-pretreated cells were ~ 130% and ~ 250% relative to the mean value measured in parental cells (Fig. 5B, all *P* < 0.05). No significant difference in non-mitochondrial OCR was observed. As expected, the Glyco ATP production was reduced while mitochondrial ATP generation was increased in DEHP-pretreated cells (Fig. 5C, D).

### DEHP pretreatment in SW620 cells leads to increased progression in mice model

**Fig. 6:**
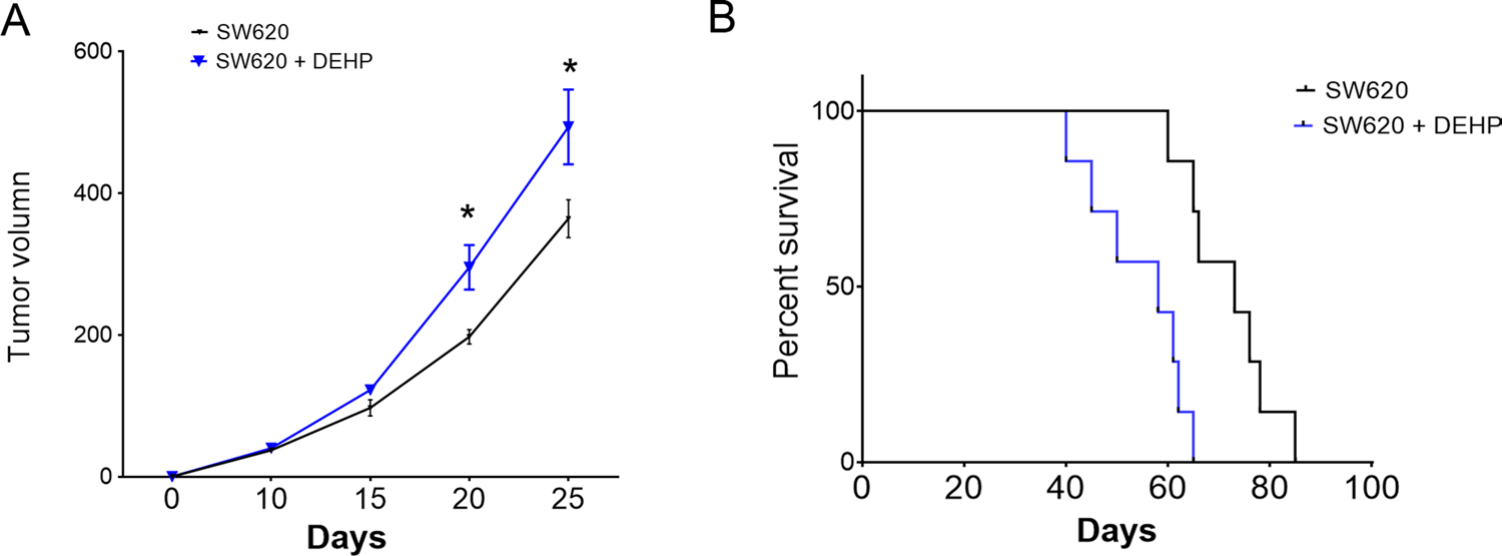
DEHP-pretreated SW620 cells showed improved progression in the mice model. **A** Total tumor volume was determined and calculated at indicated time points after the implantation of CRC cells. **B** Kaplan–Meier plot of overall survival of pelvic recurrence model. (*n* = 7, *P < 0.05)

The two groups of SW620 cells were subcutaneously injected into the flanks of nude mice to monitor the tumorigenic progression in vivo. As shown in Fig. 6, tumor volume was determined for each mouse at described time points. The tumor size was significantly larger in the DEHP-pretreated cell group since day 15 after cell implantation. The median survival of mice in the DEHP-pretreated cell group was 59.0 days, which was 69.5 in the control group implanted with parental SW620 cells.

## Discussion

DEHP is colorless, viscous, lipophilic liquid-soluble, which is the most common member of the phthalates used as plasticizers in polymer products to make plastic flexible (Wang et al. 2019). It can easily diffuse into the environment at high temperatures or when in contact with hydrophobic materials, resulting in widespread environmental pollution. Researchers have demonstrated that DEHP is toxic, which leads to embryo mortality and typical toxicity symptoms, such as tail curvature, necrosis, and cardiac edema in zebrafish with an LC50 of 0.50 ppm (Chen et al. 2014). Study has found that DEHP can cause abnormal mitochondrial ultrastructural and redox homeostasis and lead to irreversible liver damage (Camacho et al. 2020). Clinical studies also have shown that DEHP exposure can lead to decreased semen volume, reduced sperm motility, and decreased serum testosterone (Hauser et al. 2006, Spjuth et al. 2007). In addition, DEHP may disrupt sperm DNA integrity and induce sperm deformities and apoptosis. A study in rats found a statistically significant incidence of pancreatic acinar cell adenomas in animals exposed to high-dose DEHP (David et al. 2000). Given its ubiquity, the general population is likely to be exposed to DEHP. Thus, there has been increasing concern about the risk of DEHP, especially in carcinogenicity and child health.

Colorectal cancer is the 3rd most common malignancy worldwide. According to the World Health Organization database, there were more than 1.9 million new cases of colorectal cancer in the past year 2021. Despite advances in systemic therapy, the 5-year survival rate is still a mere 12.5% (Micu et al. 2020); resistance to chemotherapy remains one of the greatest challenges (Goka et al. 2019). Notably, the incidence and mortality have significantly increased in developing countries during the past decade. There are many reasons for this change in trend; among which, changes in environmental and also lifestyle exposures received more attention recently. In addition, recent studies showed that microplastics could adsorb DEHP, transport them to the gut, and cause intestinal accumulation (Deng et al. 2020, Pan et al. 2022). Therefore, it is worth studying the effect of DEHP on normal intestinal and intestinal cancer cells.

Here, we showed that long-term DEHP treatment of SW620 cells results in increased cell proliferation associated with metabolic reprogramming. Metabolic remodeling has been linked to various types of malignancies. In DEHP-pretreated SW620 cells, we observed a decrease in glycolysis activity. Interestingly, the maximal OCR and reserve capacity in DEHP-pretreated cells were drastically upregulated compared to these parental SW620 cells. Previous studies have proved that ATP-high cancer cells were phenotypically the most aggressive, with enhanced stem-like properties, showing multi-drug resistance and an increased capacity for cell migration, invasion, and spontaneous metastasis. Clinically, increasing evidence also suggests that CRC cells display upregulated oxidative phosphorylation (OXPHOS) compared with healthy colon tissues. Consistent with this theory, the DEHP-pretreated SW620 cells exhibited increased resistance to 5-FU. In previous studies, DEHP has been proven to promote the cell growth and drug resistance of different tumor cells through various signaling pathways, such as ERK, JAK2/STAT3, PI3K/Akt, etc. (Chen et al. 2013, Hsieh et al. 2022, Zhang et al. 2022). In this work, we, for the first time, focus on DEHP-mediated reprogramming of energy metabolism in intestinal cancer cells. We speculate that long-term exposure to DEHP leads to more systematic overall changes in SW620 cells rather than a particular signaling pathway, which will be investigated in our follow-up work.

Together, our data suggest that DEHP exposure results in the metabolic remodeling of colorectal cancer cells, whereby SW620 cells increase their dependence on mitochondrial OXPHOS for energy production.

## Data Availability

The data of the study can be provided by corresponding author upon reasonable request.
